# Atrial Fibrillation in Acute St-Elevation Myocardial Infarction: Clinical and Prognostic Features

**DOI:** 10.2174/157340312803760857

**Published:** 2012-11

**Authors:** Bulent Gorenek, Gulmira Kudaiberdieva

**Affiliations:** * Department of Cardiology, Faculty of Medicine, Eskisehir Osmangazi University, Eskisehir; ** Adana, Turkey

**Keywords:** Atrial fibrillation, acute myocardial infarction, prognosis, survival.

## Abstract

Atrial fibrillation (AF) is a common arrhythmia in the setting of acute coronary syndrome and acute ST-elevation myocardial infarction (STEMI). This review summarizes recent evidence on the clinical and prognostic significance of pre-existent and new-onset AF in acute STEMI patients and highlights new emerging predictors of AF development in the era of contemporary treatment.

## INTRODUCTION

Atrial fibrillation is a frequently encountered arrhythmia in the setting of acute ST-elevation myocardial infarction and acute coronary syndromes [1-7]. A population-based study [1] demonstrated that the incidence of AF in the setting of AMI tended to increase up to 13.3% during last decade. AF in the setting of AMI has a worse impact on the clinical course and prognosis of the disease. 

It could be anticipated that AF incidence, its clinical and prognostic significance might change with advancements in reperfusion strategies, including PCI and contemporary treatment with angiotensin converting enzyme inhibitors, angiotensin receptor blockers, statins and modern antithrombotic therapy in AMI patients. In parallel with the advancements in the management of AMI patients, there has been enhanced appreciation of the predictors of new- onset AF developing after acute event. Therefore, this review highlights the current knowledge on clinical and prognostic features of AF developing in the context of AMI. 

## INCIDENCE AND CLINICAL CHARACTERISTICS OF AF IN AMI (TABLE 1)

AF is encountered in 10.4-12% of cases with AMI treated by thrombolytics or primary percutaneous interventions [2-5, 8, 9], with an even higher incidence in cases with LV dysfunction [10-12]. In about 2.5-4.4% of patients treated by thrombolytics or primary PCI [2, 4] the arrhythmia existed prior to the hospital admission. This level of pre-existent AF increased to 12% in patients with LV dysfunction [10-12]. The incidence of new-onset AF, defined as the arrhythmia developing after hospital admission but during inpatient stay, was reported to vary between 6.5-7.9% in cohorts of patients included in thrombolysis or primary PCI studies [2, 4, 8, 9]. Once again the rates were increased in patients with LV dysfunction (7.2-19%) [10-12]. However these rates may under-represent the true incidence; the actual AF rates doubled when detected by long-term implantable event loop recorders (up to 16%) as compared to those recorded using ECG [13].

Analysis of the clinical features of patients with AMI and AF shows that these patients have a more adverse clinical course of their disease due to their older age, increased number of comorbidities, more frequent signs of hemodynamic compromise, worse ^*^Killip class (Appendix 1), more severe coronary artery disease and poorer perfusion after thrombolysis or primary PCI. 

In GUSTO I, a randomized trial of 4 thrombolytic regimens of streptokinase and alteplase [2], patients with AF were more likely to be older, female and have a higher HR, low BP, higher Killip class. They were also more likely to have a history of HT, prior MI, diabetes, low EF, multivessel coronary involvement, left main coronary artery disease and TIMI flow <3. Similar characteristics were also described for patients included in GISSI-3 trial where thrombolysed patients were allocated to treatment with lisinopril or lisinopril and nitrates) [9]. Here patients with AF were less likely to be treated with aspirin or beta-blockers, but more likely to be treated with digoxin, warfarin and antiarrhythmics. Patients with new-onset AF in GUSTO III (a randomized trial where AMI patients were randomised to thrombolysis with alteplase or reteplase) [8] displayed the same clinical features namely being older and female, having a history of HT, diabetes, hyperlipidemia, smoking, HF, prior MI, higher Killip class, ongoing angina and other cardiovascular disease. They were also more often being treated with antiarrhythmics, beta-blockers, ACEIs, digoxin and warfarin.

Whilst the clinical presentation of patients with AMI, AF and LV dysfunction displayed the above-mentioned characteristics, they were distinctive by having an increased occurrence of HF signs, stroke, and reduced creatinine levels [10]. Also less of the patients were treated by thrombolytics, and more of them experienced in-hospital VT and VF, cardiogenic shock and congestive HF [10]. AF patients with HF or LV dysfunction receiving treatment with ARBs and ACEIs in the VALIANT and OPTIMAAL studies [11, 12], had the similar clinical features as found in the thrombolysis trials namely older age, the presence of risk factors for coronary artery disease such as HT, smoking, dyslipidemia and a history of stroke. They were also more likely to have angina, signs of HF, higher Killip class, anterior MI localization [11], faster HR and elevated BP. Patients with prior AF were more likely to have peripheral artery disease and history of either coronary bypass surgery and/or PCI. These patients were more often treated with digoxin, amiodarone and warfarin [11], but less likely were treated with beta-blockers [12], aspirin, statins and thrombolytics, and few of them underwent primary PCI [12]. 

Among unselected patients undergoing primary PCI in the prospective OACIS study [4], patients with AF were older, often had history of prior MI and cerebrovascular disease, and more of them were hypotensive with fast HR and higher Killip class. In terms of angiographic features, the AF patients were more likely to have multivessel coronary disease and low (<3) postprocedural TIMI flow rates (79.9% vs 82.3%, with and without AF, respectively, p=0.023). In another recent prospective cohort study by Lin *et al*. [14] the AF population was frequently represented by females with history of diabetes, HT and stroke, extensive myocardial damage, higher Killip class (>3)and NYHA class, and low EF. Similarly, less of these patients had adequate postprocedural coronary perfusion flow rate (TIMI 3 flow rates 84.7% vs.93.7%, for AF and sinus rhythm groups, p=0.003). Fewer of the AF patients received stent implantation and dual antiplatelet therapy, and more of them required ECMO and intra-aortic balloon pump support. Similarly in the APEX-MI study [15], in patients bearing similar clinical characteristics those with AF had higher levels of biomarkers of myocardial damage such as creatine kinase and troponin, and higher B-type natriuretic peptide levels. In this study, few patients with AF received drug-eluting stent implantation, and the AF patients, who were inherently at a high risk for stroke, were less likely to receive triple antithrombotic therapy, including warfarin. 

## PREDICTORS OF AF DEVELOPMENT IN AMI 

As we mentioned above, new-onset AF developed during the in-hospital stay in 7.9% of patients in GUSTO I trial and in 6.5% of patients included in GUSTO III trial [2,8]. Analysis of the independent predictors of AF was presented in both of these trials. In Gusto I trial [2] the development of new-onset AF was more likely in patients of older age (risk increased by 3.2 times), with extensive myocardial damage (assessed by peak creatine kinase), with higher Killip class, fast HR and decreased systolic BP. In contrast, the risk of developing AF was found to be reduced in patients receiving reperfusion therapy with alteplase. In the GUSTO III trial [8], development of new AF whilst an inpatient was associated with older age, a history of hypertension, signs of hypotension, higher Killip class, the presence of advanced atrioventricular block or VF, new HF and treatment with digitalis and antiarrhythmics.

In OPTIMAAL study, patients with signs of HF or LV dysfunction who were receiving either ACEI or ARB, the risk of developing AF increased x1.66 for each 10-year increment of age (incidence of new onset AF=7.2%) [11]. AF development was also more common in male patients with angina pectoris, elevated HR and diastolic BP and a higher Killip class [11].

Similarly, patients who underwent primary PCI had the same predictors for developing AF as those undergoing thrombolytic therapy [4, 16]. In OACIS study, 7.7% of patients developed new AF during their inpatient stay and multivariable predictors of arrhythmia development were older age, male gender, HR>100 bpm and Killip class IV [4]. It is worth noting that the angiographic characteristics and patency of the infarct-related artery were not independent predictors of AF in this population of patients. In the more recent RISK-PCI study [16] which investigated the development of AF in patients with AMI undergoing primary PCI and receiving dual antiplatelet therapy with aspirin and clopidogrel, new-onset arrhythmia developed in 6.2% of patients. In addition to those predictors reported in the OACIS study population [4], systolic BP> 100 mmHg at admission, creatinine clearance > 60 ml/min, preprocedural infarct-related artery occlusion and postprocedural TIMI flow< 3 were identified as independent predictors of AF development [16]. 

Thus, irrespectively of treatment by thrombolytics, ACEI and ARB therapy or primary PCI as reperfusion strategy the development of AF in patients after AMI mostly depends upon their poorer clinical status, as indicated by the more frequent development of complications of AMI, hemodynamic compromise and severer comorbidities. Whether the use of drug-eluting stents and modern antithrombotic strategies aimed at better reperfusion will reduce the incidence of new-onset AF during AMI needs to be clarified as the RISK-PCI study suggested that the development of AF depended on the postprocedural TIMI flow rate [16]. 

## EMERGING PREDICTORS AND FACTORS CONTRIBUTING TO AF DEVELOPMENT IN AMI

Among the relevant mechanisms of AF development, several factors inherently related to AMI are worth mentioning: ischemia and reduced atrial perfusion, increased LV end-diastolic pressure and left atrial pressure leading to stretch-induced arrhythmias, diastolic dysfunction and abnormalities of autonomic regulation. Recently inflammation and neurohumoral factors have been shown to be associated with AF development in patients with AMI. 

Abnormal conduction of atrial impulse has been demonstrated by studies investigating the presence of increased P-wave duration and dispersion in patients with AMI and AF [17, 18]. These abnormalities were abolished after reperfusion was achieved with either thrombolysis or primary PCI [17-19], thus confirming the role of ischemia in arrhythmia development. Indeed, patients with AF in the context of AMI were more likely to have infarcts that involved the sinus and atrioventricular nodal arteries [20-22]. 

Recent prospective observational studies have confirmed the link between atrial electrical and mechanical abnormalities. In the case-controlled study, ECG – APEX MI [23], investigators compared the ECGs of 315 cases that went on to develop new- onset AF in the context of an AMI to 315 control patients who remained in sinus rhythm. Both groups were treated by primary PCI. They demonstrated that ECG signs of atrial infarction, as defined by the ^**^Liu minor criterion 1 as abnormal wide notched P wave morphology (Appendix 2), were associated with a 70% increase in the probability of new development of AF, and that this was independent of the Killip class, a history of HF and high systolic BP. Moreover, patients with new-onset AF and ECG sign of atrial infarction had a 2.43 times (95% CI 1.22 -4.84) greater 90-day mortality risk even after adjustment for other independent predictors for mortality. Further evidence of this relationship was shown in another study [24], where a significant relationship between the recurrence of AF, prolonged atrial depolarization as shown by increased P wave duration>110 ms and left atrial enlargement was found in patients with non-STEMI event. Similarly, echocardiographic indices of left atrial electromechanical dysfunction, such as prolonged total atrial activation time and increased left atrial volume, have also been shown to be independent predictors of new-onset AF in AMI patients [25].

Hemodynamic abnormalities observed in the setting of AMI, such as increases in left atrial pressure and volume, LV diastolic dysfunction [26] and the presence of a restrictive LV filling pattern [27] have been linked to the development of AF in the context of an AMI. Recently, increased LV end-diastolic pressure has been linked to increased dispersion of atrial impulse propagation [28] and this, plus the presence of mitral regurgitation [29] have also been shown to be independent predictors of new AF development after AMI. 

In terms of other emergent predictors, disturbances of the reflex autonomic heart rate control have been associated with a doubling of the likelihood of developing new AF in older patients with AMI [30]. Similarly, elevated B-type natriuretic peptide has recently been reported to be associated with new-onset AF in AMI patients [31]. The independent role of inflammation (increased C-reactive protein levels) in arrhythmia development has also been shown recently for AMI patients, specifically in those without left atrial remodeling [32, 33]. 

## PROGNOSTIC SIGNIFICANCE OF AF IN AMI (TABLE 2)

Any AF event in the setting of AMI, whether pre-existent to the admission or newly developed during hospitalization, is associated with a worse prognosis in patients undergoing thrombolysis or primary PCI, or being treated with ACEI and ARBs. Analysis of the impact of AF on prognosis of patients included in RCTs receiving thrombolysis [2, 8, 9] showed that although arrhythmia often developed secondary to AMI complications, it had an independent value in predicting of short- and long- term mortality. 

In GUSTO I trial [2], 2.5% (1026) of patients had AF on admission, 7.9% developed AF after enrollment and 89.6% (36611) had no signs of the arrhythmia. The in-hospital (5.8% vs 13.8%, p=0.0001,) 30-day (6.1% vs 14.3%, p=0.0001) and 1-year (8.4% vs 21.5%, p=0.0001) mortality rates were significantly higher in any AF patients as compared to those without AF. Indeed, the adjusted risk for 30-day mortality was 1.3 (95% CI 1.2-1.4) times higher for patients with any AF, and 1.4 times higher for patients with new onset of arrhythmia (95%CI 1.3-1.5), even though the mortality risk for AF on admission was not significant. 

In GUSTO III trial (8), among 13858 patients with sinus rhythm at enrollment, 6.5% (906) developed new AF during their inpatient stay. No difference in survival was found between patients that developed AF early in their clinical pathway (<48 hours) compared to those who developed the arrhythmia later on (>48 hours). Patients with AF had a 1.49 (95% CI, 1.17-1.89) times higher probability of dying irrespectively of other independent predictors of mortality such as ischemia and reinfarction, HF, cardiogenic shock, life-threatening arrhythmias or severe bleeding. The independent predictive value of new- onset AF on mortality was sustained out to 1 year after an acute event (OR- 1.64 95% CI, 1.35-2.01). 

New-onset AF is also an independent predictor of long-term (up to 4 years) mortality in patients treated with thrombolysis and ACEI [9]. In the GISSI -3 trial, overall 1386 of 17944 (7.8%) unselected patients with AMI enrolled during first 24 hours of presentation and receiving optimal treatment with thrombolytics (72%) and randomized for treatment arms with ACEI lisinopril, lisinopril/nitrates and nitrates alone developed AF during their in-hospital stay [9]. In this trial patients with AF had a 1.98 (95%CI 1.67-2.34) times increased inpatient mortality risk, which persisted out to 6-months (RR 1.81 95%CI 1.48-2.23) and throughout the 4-year trial period (RR 1.78 95% CI 1.6-1.99). Other independent predictors of mortality in this cohort were advanced age, Killip class 2-3, LV dilatation and the presence of congestive HF. When the patients with AF were subdivided into the subgroups according to the time of arrhythmia development, it was revealed that patients who developed AF after day 1 had higher inpatient mortality as compared with those who developed AF on or before day 1 (18.9%, vs 13.3%, OR 1.50, 95% CI 1.08 to 2.08). Interestingly, no differences in long-term mortality were observed between these groups.

In AMI patients with AF, signs of LV dysfunction or HF adversely affected not only short- and long-term mortality, but also other cardiovascular outcomes, including stroke, HF and recurrent MI [10-12, 34, 35]. 

AF/Afl was seen in every 5^th^ patient (21%) with large AMI and LV dysfunction that were screened for inclusion in TRACE study [10]. The presence of AF/Afl predicted both the long-term, 5-year mortality and the incidence of stroke, and did so independently of LV dysfunction (estimated by wall motion index) and thrombolytic treatment. However, the long-term mortality in these patients was primarily dependent on the severity and progression of underlying disease [35]. 

The TRACE study screening database incorporates 6767 patients with AMI, of whom 75% were treated by thrombolytics [10]. From this initial cohort, 1577 of them were then further selected for randomized treatment with either trandolapril or placebo. AF/Afl was recorded during the inpatient stay in 21% of patients; 3.9% of patients had pre-existent arrhythmia, and 5% of patients had AF/Afl sustained during their whole hospitalization. In this study, inpatient (18% vs 9%, p<0.001) and 5-year mortality (56% vs. 34%, p<0.001) rates were significantly higher for patients with AF/Afl compared to patients without AF. Patients who developed the arrhythmia during hospitalization had a 1.5 times higher probability of dying during their hospital stay (adjusted for left ventricular dysfunction and other baseline variables OR=1.5, 95% CI 1.2–1.9, p<0.001), whilst those with pre-existing AF/Afl did not. However, at 5-year follow-up both groups of patients (i.e. those with AF prior to admission and those with newly developed AF) had a similar increase in risk of mortality (RR-1.3, 95% 1.1-1.5, RR -1.4, 95%CI 1.2-1.7, respectively). Patients in whom the arrhythmia was sustained arrhythmia throughout their hospital stay had higher relative risk of mortality as compared with those who had intermittent episodes of arrhythmia.

In the randomized placebo-controlled arm of the TRACE study [34], 1577 patients in sinus rhythm during enrollment were further randomized to either treatment with trandolapril (790 patients) or placebo (787 patients). AF developed in 5.3% of patients in the placebo group and 2.8% in the trandolapril group (p<0.05). It was also shown that treatment with trandolapril reduced the risk of developing of AF by 55% (RR=0.45, 95%CI -0.26-0.76, p=0.01) during 2 to 4-year of follow-up period in those patients with AMI and LV systolic dysfunction (WMI≤1.2 or EF ≤36%)[34]. 

Analysis of the mode of death in patients with AF in the TRACE study [35] showed that the adjusted risk was increased by 1.31 times (1.07-1.6) for SCD and similarly by 1.43 times for non SCD and that this was independent of age, gender, EF, previous MI, HF, angina pectoris, diabetes, hypertension or the presences of bundle branch block. There were no significant differences in the risk of SCD among the subgroups of hypertension, diabetes, low EF, presence of VT/VF or QRS width; all subgroups being associated with an increased risk of SCD. Analysis of the beneficial effects of trandolapril revealed that it reduced 3-year total mortality by 30% (RR=0.70, 95% CI: 0.51–0.95) and cardiovascular death by 33% (RR =0.67, 95% CI: 0.47–0.96), but that the risk reduction for SCD was insignificant (p=0.27). Therefore it was concluded that although AF/AFL may facilitate the initiation of ventricular tachyarrhythmias, multiple mechanisms and severity of underlying cardiovascular disease also be fundamental to the cause of mortality, and thus that this group of patients may not benefit from antiarrhythmic therapy. 

The negative prognostic value of AF was also shown for patients with AMI and signs of HF and LV dysfunction, treated with ARBs/ACEIs [11, 12]. It was demonstrated that AF in these patients, similar to patients with uncomplicated AMI, was associated with a worse short and long-term prognosis, including increased risk of stroke, HF, MI and SCD.

Analysis of the results of OPTIMAAL [11], a randomized active-controlled study investigating the effects of the ARB losartan vs the ACEI captopril on outcomes in 5477 patients with AMI and LV dysfunction (LVEF <40% or LVEDD>6.5 mm) 54.4% of whom were treated with thrombolytics, revealed that 655 patients were admitted with prior AF (12%) and 345 (7.2%) developed AF during the enrollment period – i.e the first 10 days of MI. It was found that preexistent AF was associated with significant rise in 3-year stroke, total mortality, cardiovascular death and re-hospitalization rates (adjusted HRs - 1.77, 1.32, 1.31 and 1.14, p=0.001, p=0.003, p<0.001, p<0.012, respectively). During the first 30 days after MI, new-onset AF markedly increased the risk of developing a stroke by 14.6 times (95%CI 5.87-36.3), cardiovascular death by 3.98 (2.05–7.74) and total mortality by 3.83 times (95%CI 1.97-7.43). This prognostic value was sustained until the 3rd year of trial with double the risk of stroke (HR- 2.29, 95%CI1.43–3.68), total mortality (HR-1.82, 95%CI1.39–2.39) and cardiovascular death (HR -1.91, 95%1.41–2.58). The AF rates did not differ between treatment randomization groups: losartan (7.3%) or captopril (7.0%) groups.

The VALIANT study [12], included 14703 (of whom 14660 were included in the final analysis) patients with AMI and signs of HF and LV systolic dysfunction. Among this cohort 35.1% received thrombolytic therapy and 14.8% underwent primary PCI. All the patients were randomized to either treatment with valsartan, captopril or valsartan plus captopril. Prior AF was found in 339 patients (2.3%) and new – onset AF developed in 1812 (12.3%) patients. During the 3 years follow-up patients with any AF had 1.3 (95%CI 1.19-1.43) times higher risk of dying as compared with patients without AF. Among them, likelihood of survival was worse for patients with new-onset AF was 1.32 times higher (1.2-1.45), as well as for those with prior AF (HR 1.25, 95%CI 1.03-1.52) compared to patients without AF. Also during the 3 years of trial, new-onset AF was associated with reduced survival free from the composite outcome that included cardiovascular death, HF, MI, stroke and SCD (HR 1.21, 95%CI 1.12– 1.31) irrespectively of clinical, laboratory variables and treatment. The mortality rates of AF patients in VALIANT trial did not differ between treatment groups: valsartan, captopril and valsartan plus captopril.

Recently, underdiagnosed asymptomatic AF, that comprises the vast majority of all episodes detected by implantable loop recorders, was found to be important in the prediction of cardiovascular morbidity and mortality [13]. The CARISMA study [13] included 271 patients after AMI with LV dysfunction (EF<40%) who were treated either by thrombolytics or PCI and 96% of whom received ACEI/-ARB and beta-blocker therapy, anticoagulant and/or antiplatelet therapy. It was found out that new-onset AF rate, as detected by implantable loop recorder implanted 2 months after MI, was as twice high (16%) as compared the incidence previously reported in studies, and that about 90% of the episodes were asymptomatic. The authors demonstrated that AF >30sec in duration was a significant predictor of major cardiovascular events (reinfarction, stroke, hospitalizations for heart failure and cardiac death) during 2 –year follow up period (HR 2.73, 95%CI 1.35–5.50, p=0 .005) and that this was independent of wide QRS duration and previous history of MI. 

In patients undergoing primary PCI, AF was not found to be an independent determinant of inpatient mortality, but remained a significant predictor of 30-day mortality [14, 16], 90-day outcomes [15] and 1-year mortality [4]. This could be partly attributable to the fact that better TIMI-3 flow rates were achieved using PCI and adjunct antithrombotic therapy, and the use of contemporary treatment with ACEIs [9,34], ARBs [11, 12], beta-blockers [36] and statins [37, 38] all of which have been shown to reduce the risk of AF development especially in patients with higher Killip, NYHA class and LV dysfunction. Similar to results of the TRACE study, analysis of the mode of death 1-year after primary PCI [35] showed that major determinants of mortality were cardiovascular death and pump failure [4] but not arrhythmic causes. Progression of LV dysfunction and major comorbidities were the main contributors to mortality in patients with AF that underwent primary PCI. 

In the OACIS study [4] 2475 out of 3614 consecutive patients with AMI underwent primary PCI within first 24 hours of disease onset. Among them 297 patients (12%) had AF/Afl, including 4.4% (107 pts) who had the arrhythmia on admission and 7.7% (190 pts) who developed AF/Afl during hospitalization. Patients with AF/Afl were more likely to die, to experience cardiogenic shock, HF or to develop a stroke during their hospitalization. However, after adjustment for clinical variables, AF/Afl was not predictive for these inpatient events. After 1 -year follow- up the mortality rates were higher in those patients who developed AF during their hospitalization compared to those who remained in sinus rhythm (3.6% vs 1.3%, p=0.003). New-onset AF, but not prior AF, was found to be an independent predictor of 1-year mortality (HR=3.05, 95%CI 1.22-7.62, p=0.017). 

In a recent study by Lin *et al*. [14], of 783 patients with AMI, who underwent primary PCI and received contemporary treatment including ACEI, ARB and beta-blockers, AF was detected in 10.9% of the patients, including 52 patients who had AF at enrollment and 33 patients who developed AF during their hospital stay. AF was a significant univariate, but not a multivariate predictor of 30-day mortality (12.9% vs 4.7%, HR – 2.816, 95%CI 1.423-5.572, p=0.003). The only significant multivariate predictors of 30-mortality were Killip class>3 and low EF.

In the RISK-PCI study [16] the rate of onset of new AF was 6.2% among the 2096 patients receiving dual antiplatelet therapy and undergoing primary PCI. In this study, the 30-day mortality and major cardiovascular events rates were 2.39-2.67 fold higher for patients with new onset AF. There was also a trend toward higher rates of target vessel revascularization due to ischemia in AF group. 

In a recent analysis of the APEX-MI study [15], which included 5745 AMI patients treated with primary PCI and receiving comprehensive double and triple antithrombotic therapy, the new onset AF rate was 6.3%. New onset AF was associated with inpatient complications such as VT/VF, atrioventricular block, asystole, cardiac arrest, hypotension, and cardiac tamponade. AF also predicted the 90- day mortality (HR-1.81, 95%CI 1.06-3.09) independently of other confounding variables. Patients with new onset AF were also at increased risk of developing shock (x3.81), congestive HF (x2.66) and stroke (x2.98) during the 90- day period after AMI event. The important result of this study was that warfarin use in AF patients was accompanied by a reduced risk of 90-day mortality and stroke. Triple antithrombotic therapy in this trial was associated with lower mortality and stroke rates. 

## TREATMENT OF AF IN THE SETTING OF STEMI 

As the new-onset AF depends more on the underlying causes and comorbidities, and mode of death is not predominantly due to arrhythmias, treatment of underlying causes gains utmost priority.

Analysis of GUSTO III trial data [39] showed that use of class I antiarrhythmics for conversion of AF in AMI patients was associated with a trend to lower mortality, while amiodarone and electrical cardioversion had no impact on mortality rates. On the other hand, antiarrhythmics increased the risk of mortality in AF patients with signs of HF. Recent analysis of the VALIANT study data [40], demonstrated that when a rhythm control strategy was used in 371 of the patients with AF after AMI (87.3% of patients received amiodarone, 14.8% - other antiarrhythmics) this was associated with 2- fold excess risk of early mortality during first 45 days of disease (HR= 1.9, 95% CI 1.2 - 3.0, p=0.004) compared to employing a rate control strategy group (where about 85% of these 760 patients were on beta-blockers). 

The use of beta-blockers (carvedilol) in patients with AMI [36] has been shown to reduce risk of AF development. Similarly, treatment with ACEI in patients with and without LV dysfunction has been associated with 24% and 55% reduction in the risk of AF development during follow -up period [8, 34]. Several studies have shown that patients receiving treatment with statins prior to admission were less likely to develop further AF [37]. Indeed, in the recent prospective FAST-AMI study [38] multivariate analysis demonstrated that early therapy with statins reduced the risk of new AF development in STEMI and NSTEMI patients by 36% (95% CI 0.45 - 0.92, p=0.017) independent of confounding factors. On the other hand, analysis of the PROVE IT–TIMI 22 and A -to -Z randomized controlled trials` data did not show a benefit in terms of AF risk reduction with higher doses of atorvastatin or simvastatin, despite demonstrating a tendency to increased C-reactive protein levels in patients with AF [41].

Current guidelines [42, 43] on the management of STEMI and AF recommend the use of beta-blockers (if no HF, bronchospasm or block) or non-dihidropyridine calcium antagonists for the rate control as a class I indication. Similarly, as a class I recommendation, amiodarone use is indicated for slowing rapid ventricular response and to improve LV function. As a class IIB recommendation, in severe LV dysfunction digoxin may be preferred as the rate control agent of choice. In case of hemodynamic instability, ongoing ischemia or persistent fast rates despite pharmacological rate control therapy electrical cardioversion is recommended as a class I indication. It is recommended not to use class III antiarrhythmics agents, but rather one must treat the underlying complications and disease. Proper anticoagulation is also indicated. 

## CONCLUSION

AF, especially the new-onset form of the arrhythmia, usually develops secondary to complications in the context of STEMI. However, AF is an independent predictor of short and long-term unfavorable outcomes in this category of patients irrespectively of the reperfusion strategy. The mechanisms underlying AF development in the setting of STEMI are multifactorial and depend on the severity of the main disease. Clinical predictors for AF development remain consistent throughout all the studies on thrombolysis, primary PCI, ACEI and ARBs. However analysis of the predictors of new AF development in patients treated with drug-eluting and bare-metal stents with adjunctive modern antithrombotic therapy need to be addressed. Treatment of underlying cardiovascular causes and HF, the main components of mode of death in the 1st year post AMI, should be targeted as a priority. Effective prevention of new AF development might be achieved by defining the role of emerging novel predictors of arrhythmia. 

## Figures and Tables

**Table 1.** Study Description and Incidence of Atrial Fibrillation in STEMI Patients

Study	Pts, n	Design	Inclusion criteria	Treatment	Trial Period	Any AF, %	Prior AF, %	New-Onset/In- Hospital AF, %
GUSTO I^2^	40891	RCT	STEMI	Thrombolysis streptokinase vs alteplase	1 year	10.4%	2.5%	7.9%
GUSTO III^8^	13858	RCT	STEMI	Thrombolysis alteplase vs reteplase	1 year	-	-	6.5%
GISSI^9^	17944	RCT	STEMI	Thrombolysis 72% lisinopril/lisinopril+nitrates/nitrates	4 years	-	-	7.8%
TRACE^10^	6776	RCT Pre-enrolment	STEMI LV dysfunction	Thrombolysis 75% of patients	5 years	-	3.9%	21%
OPTIMAAL^11^	5477	RCT	STEMI HF and LV dysfunction (EF<40% or LVED>=65)	Thrombolytics- 54.4% Captopril vs losartan	3 years	-	12%	7.2%
VALIANT^12^	14703	RCT	STEMI Radiological or clinical HF and/or LV dysfunction	Thrombolytics 35.1%, primary PCI 14.8% Captorpil, valsartan or both	3 years	-	2.3%	12.3%
OACIS^4^	2475	Observational cohort study	STEMI	Primary PCI	1 year	12%	4.3%	7.7%
APEX-MI^15^	5745	Observational cohort	STEMI	Primary PCI, dual and triple antithrombotic therapy		11%	4.8%	6.3%

**Table 2.** Prognostic significance of AF in STEMI patients

Study	Risk of mortality	
	In-hospital/30-day/90-day	≥1-year
** GUSTO I^2^**		
Any AF	30-day *OR 1.3 (1.2-1.4)	1-year n.a. Kaplan-Meier estimates: 21.5 vs 8.4%, p<0.001
Prior AF	30-day *ns	1-year n.a. Kaplan-Meier estimates: 22.2 vs 8.4%,p<0.001
New-onset AF	30-day *OR 1.4 (1.3-1.5)	1-year n.a. Kaplan-Meier estimates: 21.2 vs 8.4%,p<0.001
** GUSTO III^8^**		
New-onset AF	30-day **OR 1.49 (1.17-1.89)	1-year **OR 1.64 (1.35-2.01)
** GISSI^9^**		
New-onset AF	In-hospital *RR 1.98 (1.67-2.34)	4-year *RR 1.78 ( 1.6-1.99)
** TRACE^10^**		
Any AF	In-hospital *OR 1.5 (1.2-1.9)	5-year *RR 1.3 (1.2-1.4)
Prior AF	In-hospital *OR 1.2 (0.8-1.9) n.s.	5-year *RR 1.3 (1.2-1.4)
New-onset AF	In-hospital *OR 1.5 ( 1.2-1.9)	5-year *RR 1.4 (1.2-1.7)
**OPTIMAAL**^11^		
Prior AF	30-day n.s.	3-year *HR 1.32 (1.13-1.56)
New-onset AF	30-day *HR 3.83(1.97-7.43)	3-year *HR 1.82 (1.39-2.39)
**VALIANT**^12^		
Any AF	-	3-year *HR 1.3 (1.19-1.43)
Prior AF	-	3-year *HR1.25 (1.03-1.54)
New-onset AF	-	3-year *HR1.32 (1.2-1.45)
**OACIS^4^**		
Any AF	In-hospital *HR 1.42 (0.88-2.31) n.s.	1-year *HR 1.64 (1.05-2.55)
Prior AF	In-hospital *n.s.	1-year *HR 1.87 (0.45-7.52) n.s.
New-onset AF	In-hospital *n.s.	1-year *HR 3.04(1.24-7.48)
**APEX-MI^15^**		
New onset AF	90-day HR**1.81(1.06-3.09)	-

* adjustment for baseline clinical variables, treatment

** adjustment for baseline clinical variables and in-hospital complications pre-AF

95% confidence intervals are presented in brackets, HR – hazard ratio, OR – odds ratio, RR – relative risk, n.a. – not available, n.s. – not significant

## Footnotes

***APPENDIX 1:**

**Table:** Killip classification of patients with acute myocardial infraction (modified)

Class I – patients are free of rales and a third heart sound
Class II – patients have rales but only to a mild to moderate degree (<50% of lung fields) and they may or may not have an S3.
Class III – patients have rales >50% of each lung field and frequently have pulmonary edema
Class IV – patients are in cardiogenic shock.

(Killip T 3rd, Kimball JT. Treatment of myocardial infarction in a coronary care unit. A two year experience with 250 patients. Am J Cardiol 1967; 20: 457-64.; Antman EM. ST-segment elevation myocardial infarction: pathology, pathophysiology, and clinical features. In: Bonow RO, Mann DL, Zipes DP, Libby P, Braunwald E, eds. Braunwald's heart disease: a textbook of cardiovascular medicine. 9th ed. Philadelphia, PA : Elsevier/Saunders 2012; pp. 1087-1110.)

****APPENDIX 2:**

**Table:** Liu et al. electrocardiogram criteria for atrial infarction.

**Major criteria**
1 0.5-mm PR segment elevation in lead V5 and V6 with reciprocal depression in leads V1 and V2
2 0.5-mm PR elevation in lead I with depression in leads II or III
3 Depression of the PR segment of more than 1.5 mm. in precordial leads and 1.2 mm. in leads I, II, and III in the presence of any form of atrial arrhythmia.
**Minor criteria**
1 Abnormal P waves: M-shaped, W-shaped, irregular or notched
2 Depression of the PR segment of small amplitude without elevation of this segment in other leads

(Liu CK, Greenspan G, Piccirillo RT. Atrial infarction of the heart. Circulation 1961; 23:331-8.)

## LIST OF ABBREVIATIONS

ACEI: = Angiotensin converting enzyme inhibitors
AF: = Atrial fibrillation
Afl: = Atrial flutter
AMI: = Acute myocardial infarction
ARB: = Angiotensin receptor blockers
BP: = Blood pressure
ECG: = Electrocardiogram
EF: = Ejection fraction
HF: = Heart failure
HR: = Heart rate
HT: = Hypertension
LV: = Left ventricular
LVEDD: = Left ventricular end-diastolic dimension
MI: = Myocardial infarction
PCI: = Percutaneous coronary intervention
SCD: = Sudden cardiac death
STEMI: = ST-elevation myocardial infarction
NSTEMI: = Non –ST- elevation myocardial infarction
VF: = Ventricular fibrillation
VT: = Ventricular tachycardia
WMI: = Wall motion index

## TRIALS;

A to Z: = Aggrastat to Zocor
APEX-AMI: = Assessment of Pexelizumab in Acute Myocardial Infarction
CARISMA: = Cardiac Arrhythmias and Risk Stratification after Acute Myocardial Infarction
CAPRICORN: = CArvedilol Post-infaRct survIval COntRolled evaluation
FAST –MI: = French registry of Acute ST-elevation and non-ST-elevation Myocardial Infarction
GISSI -3: = Effects of Lisinopril and Transdermal Glycerol Trinitrate Singly and Together on 6-week Mortality and Ventricular Function after AMI
GUSTO – I: = Global Utilization of Streptokinase and TPA for Occluded Arteries-I
GUSTO-III: = Global Utilization of Streptokinase and TPA for Occluded Arteries-III
OACIS: = Osaka Acute Coronary Insufficiency Study
OPTIMAAL: = The Optimal Trial in Myocardial Infarction with the Angiotensin II Antagonist Losartan
PROVE IT–TIMI 22: = Pravastatin or Atorvastatin Evaluation and Infection Therapy–Thrombolysis in Myocardial Infarction 22
TRACE: = The Trandolapril Cardiac Evaluation Study
VALIANT: = Valsartan in Acute Myocardial Infarction Trial Investigators
